# Redo ventral rectopexy: is it worthwhile?

**DOI:** 10.1007/s10151-020-02369-5

**Published:** 2020-11-05

**Authors:** K. E. Laitakari, J. K. Mäkelä-Kaikkonen, M. Kairaluoma, A. Junttila, J. Kössi, P. Ohtonen, T. T. Rautio

**Affiliations:** 1 Division of Gastroenterology, Department of Surgery, Oulu University Hospital, Oulu, Finland; 2 Medical Research Centre Oulu, Centre of Surgical Research, University of Oulu, Oulu, Finland; 3 Department of Surgery, Keski-Suomi Central Hospital, Jyväskylä, Finland; 4 Department of Surgery, Päijät-Häme Central Hospital, Lahti, Finland

**Keywords:** Rectal prolapse, Ventral rectopexy, Mesh, Redo surgery

## Abstract

**Background:**

Minimally invasive ventral mesh rectopexy (VMR) is a widely used surgical treatment for posterior pelvic organ prolapse; however, evidence of the utility of revisional surgery is lacking. Our aim was to assess the technical details, safety and outcomes of redo minimally invasive VMR for patients with external rectal prolapse (ERP) recurrence or relapsed symptoms of internal rectal prolapse (IRP).

**Methods:**

This is a retrospective cohort study of patients with recurrent ERP or symptomatic IRP who underwent redo minimally invasive VMR between 2011 and 2016. The study was conducted at three hospitals in Finland. Data collected retrospectively included patient demographics, in addition to perioperative and short-term postoperative findings. At follow-up, all living patients were sent a questionnaire concerning postoperative disease-related symptoms and quality of life.

**Results:**

A total of 43 redo minimally invasive VMR were performed during the study period. The indication for reoperation was recurrent ERP in 22 patients and relapsed symptoms of IRP in 21 patients. In most operations (62.8%), the previously used mesh was left in situ and a new one was placed. Ten (23.3%) patients experienced complications, including 2 (4.7%) mesh-related complications. The recurrence rate was 4.5% for ERP. Three patients out of 43 were reoperated on for various reasons. One patient required postoperative laparoscopic hematoma evacuation. Patients operated on for recurrent ERP seemed to benefit more from the reoperation.

**Conclusions:**

Minimally invasive redo VMR appears to be a safe and effective procedure for treating posterior pelvic floor dysfunction with acceptable recurrence and reoperation rates.

## Introduction

Due to a lack of sufficient evidence, there are no broadly adopted recommendations about the use of either perineal or transabdominal techniques or what kind of procedure is the most effective for rectopexy when treating primary rectal prolapse [1]. Despite this, laparoscopic ventral mesh rectopexy (LVMR) has become the treatment of choice for external rectal prolapse (ERP) in Europe [2]. LVMR has also been offered to patients with internal rectal prolapse (IRP) and other pelvic anatomical disorders involving obstructed defecation symptoms (ODS) or incontinence, although there is only level 3 evidence to support this approach [3]. Patient selection is essential, since the correlation between anatomical changes and functional symptoms is not always consistent. After a primary operation, about 20% of patients with ODS and 25% of patients suffering from anal incontinence have persistent symptoms and still seek help [4–6]. On the other hand, in some patients, the symptoms have subsided even though the pelvic floor anatomy has not been restored.

Recurrence rates of ERP, IRP and/or rectocele after ventral rectopexy (VR) have been reported to vary from 0 to 14.3% and are shown to be comparable between LVMR and robot-assisted ventral mesh rectopexy (RVMR) techniques [6–10]. Some risk factors for recurrence have been proposed. A recent meta-analysis that included 17 studies and 1242 patients revealed male sex and length of the mesh as potential recurrence factors for rectal prolapse after LVMR [9]. In addition, age over 70 years, poor preoperative continence, a prolonged pudendal nerve terminal motor latency and benign joint hypermobility syndrome may predict recurrence after LVMR for various indications [11, 12].

There are no guidelines on what to do after recurrence of ERP or symptoms caused by recurrent enterocele or IRP. A systematic review 5 years ago on ERP reoperations failed to develop a treatment algorithm for recurrent rectal prolapse [13]. In addition, the recommendations about the operative technique for redo VR are missing because of a lack of evidence. A panel of experts has stated that revisional surgery in patients with recurrence should be performed in specialised centres after a detailed reassessment [14]. However, they could not comment on other aspects of redo surgery in their consensus report, since there was, and still is, an urgent need for guidance on clinical decision-making after unsuccessful LVMR. There are only two small studies that report the results of LVMR or RVMR after a primary ERP operation using a variety of surgical techniques [15, 16].

The aim of our study was to evaluate and compare the technical details, safety and outcomes of redo minimally invasive VMR for either recurrent external or relapsed symptomatic internal rectal prolapse.

## Materials and methods

### Study population and data collection

All consecutive patients with recurrent external or relapsed symptomatic IRP who underwent redo VR with minimally invasive technique between 2011 and 2016 were included in the study. The RVMR procedures were performed in one academic tertiary referral hospital (Oulu University Hospital) and LVMR operations in two central hospitals (Keski-Suomi Central Hospital and Päijät-Häme Central Hospital) in Finland. The indications for surgery and the follow-up schedule were determined according to each centre’s practice. Data concerning patient characteristics, perioperative details and short-term postoperative outcomes were collected retrospectively using electronic patient files. For the follow-up, a questionnaire concerning postoperative disease-related symptoms and quality of life were sent to all patients who were still alive. All gathered data were collected in one database. The study protocol was approved by the Ethics Committee of the Oulu University Hospital.

### Outcomes

Patient characteristics and preoperative variables recorded included age, sex, body mass index, American Society of Anesthesiologists (ASA) class, number of underlying medical conditions, diagnosis for redo surgery, indications/symptoms for primary and redo operations, previously performed pelvic surgery, previously performed hysterectomy and any other abdominal surgery, primary operation technique and mesh used, preoperative imaging and time from primary operation to redo surgery.

Perioperative data included the following: operation time, operating theatre time, bleeding, conversion, findings during redo operation, redo operation technique, mesh used in redo operations, caudal fixation of mesh, number of stiches to rectum, use of vaginal stiches and suturing technique. Short-term postoperative data included length of hospital stay, complications, surgery because of complications and reoperations due to other reasons. Long-term functional and quality of life outcomes were assessed by questionnaires sent to all patients who were alive at follow-up. The follow-up time was defined as the time from redo operation to questionnaire response date. The functional outcome evaluation included Wexner score for faecal incontinence [17] and ODS score [18]. A Wexner score > 9 and an ODS score > 20 were considered to indicate significant ongoing symptoms. Discomfort with incontinence/ODS changed by the operation and the impact of surgery on quality of life (QoL) were assessed on a visual analogue scale ([VAS], 0–100). Significant defecatory symptom relief was defined if the reported postoperative VAS figure was within the limits of 61–100. Patients were asked about postoperative satisfaction with sexual life, possible de novo symptoms (urinary incontinence, urinary retention, pelvic pain) and satisfaction with the redo operation.

### Surgical technique

The surgical procedures were primarily carried out as described by D’Hoore and Penninckx [19] with minor modifications, as described earlier [6]. Non-absorbable braided sutures were used to attach the mesh to the bowel and levator muscles. Redo operations were performed using the procedure chosen by the surgeons according to their experience and preoperative findings. The data of the technique used, and its details were carefully collected from the operation reports and from the surgeons who performed redo rectopexies.

### Statistical analysis

Analyses were performed using SPSS for Windows (IBM Corp. Released 2017. IBM SPSS Statistics for Windows, Version 25.0. Armonk, NY, USA: IBM Corp.). Summary statistics are presented as mean with standard deviation unless other stated. Between-group comparison for continuous data was analysed using Student’s *t* test or Welch test; the latter was used if assumption of equal variances did not hold. Categorical data were analysed using Fisher’s exact test. Two-tailed *p* values are presented.

## Results

In total, 43 consecutive patients (41 female and 2 male) underwent redo LVMR or RVMR from 2011 to 2016 in the 3 participating hospitals (Oulu: 8, Jyväskylä: 22, Lahti: 13). Two surgeons performed all 35 LVMR and another 3 surgeons did the 8 robotic operations. Baseline data are presented in Table 1. The results of the operations were divided into two groups according to anatomical diagnosis for the LVMR or RVMR: 22 operations (51.2%) were performed because of ERP and 21 operations (48.8%) for IRP. Patients with IRP more often suffered recurrent obstructed defecation than patients with ERP (76.2% vs. 4.5%, *p* < 0.001). In other respects, the ERP and IRP groups did not differ in terms of redo operation indication. In the IRP group, hysterectomy was done more frequently (70.0% vs. 35.3%, *p* = 0.05) than in the ERP group.

**Table 1:** Patient characteristics

	All (*n* = 43)	ERP (*n* = 22)	IRP (*n* = 21)	*p*
Age (years) mean (SD) Range	67.4 (14.2) (28–91)	69.5 (16.3) (28–91)	65.3 (11.8) (29–83)	0.34
Women [*n* (%)]	41 (95.3)	20 (90.9)	21 (100)	0.49
Body mass index [kg/m^2^ mean (SD)]	25.7 (4.8)	25.2 (4.5)	26.3 (5.2)	0.48
ASA class [*n* (%)]				
1	15 (34.9)	6 (27.3)	9 (42.9)	0.48
2	16 (37.2)	8 (36.4)	8 (38.1)	
3	10 (23.3)	6 (27.3)	4 (19.0)	
4	2 (4.7)	2 (9.1)	0 (0)	
Medical conditions [*n* (%)]				
0	7 (16.3)	3 (13.6)	4 (19.0)	0.29
1	15 (34.9)	7 (31.8)	8 (38.1)	
2	9 (20.9)	4 (18.2)	5 (23.8)	
3 or more	12 (28.0)	8 (36.3)	4 (19.1)	
Indication/predominant symptom [*n* (%)]				
Prolapse	17 (39.5)	17 (77.3)	0 (0)	< 0.001
Prolapse with incontinence	3 (7.0)	3 (13.4)	0 (0)	0.23
ODS	17 (39.5)	1 (4.5)	16 (76.2)	< 0.001
Incontinence	4 (9.3)	1 (4.5)	3 (14.3)	0.35
Bulge feeling	2 (4.7)	0 (0)	2 (9.5)	0.23
Previous pelvic surgery [*n* (%)]				0.75
No	33 (76.7)	16 (72.7)	17 (81.0)	
Posterior colporrhaphy	4 (9.3)	2 (9.1)	2 (9.5)	
Posterior vaginal mesh	1 (2.3)	1 (4.5)	0 (0)	
Anterior colporrhaphy	5 (11.6)	3 (13.6)	2 (9.5)	
Previous hysterectomy [*n* (%)]	20 (54.1)	6 (35.3)	14 (70.0)	0.05
Previous abdominal surgery [*n* (%)]				0.89
0	2 (4.7)	1 (4.5)	1 (4.8)	
1	28 (65.1)	15 (68.2)	13 (61.9)	
2 or more	13 (30.3)	6 (27.2)	7 (33.4)	
Preoperative imaging [*n* (%)]				< 0.001
No	19 (44.2)	16 (71.7)	3 (14.3)	
Defecography	18 (41.9)	5 (22.7)	13 (61.9)	
MRI defecography	6 (14.0)	1 (4.5)	5 (23.8)	
Time from primary to redo surgery [mean (SD), years]	3.5 (2.2)	3.7 (2.2)	3.4 (2.1)	0.58

Nominal variables are presented as counts and percentages (in parentheses). Continuous variables are presented as means and standard deviations

*ERP* external rectal prolapse, *IRP* internal rectal prolapse, *BMI* body mass index, *ASA* American Society of Anesthesiologists, *ODS* obstructed defecation syndrome, *MRI* magnetic resonance imaging

**Fig. 1:**
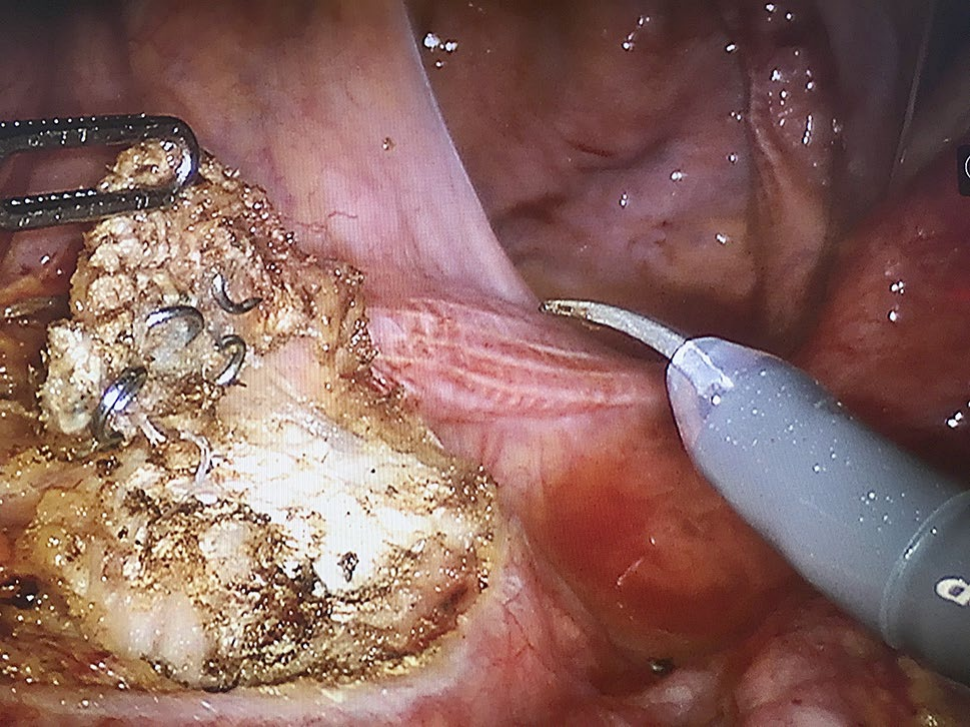
Removal of previous mesh

**Fig. 2:**
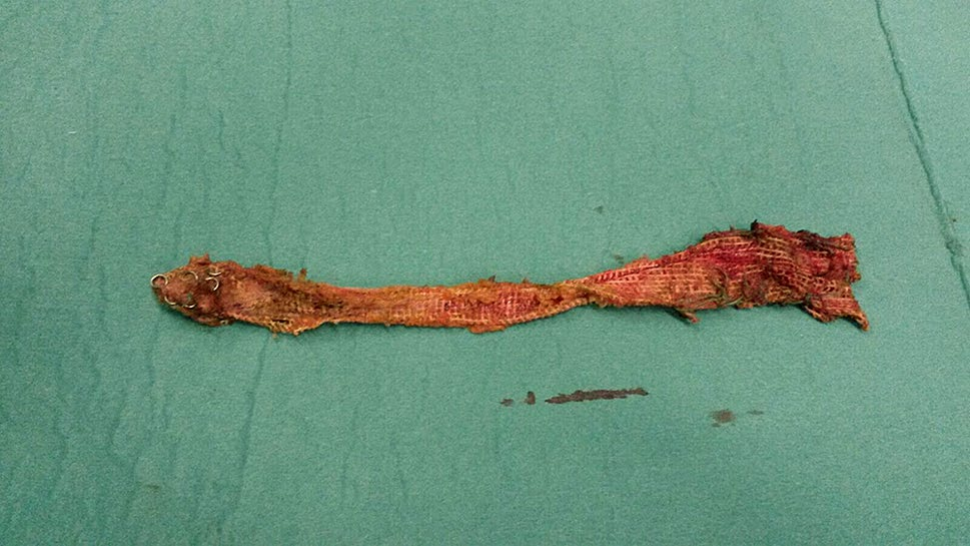
The completely removed mesh

The intraoperative findings and technical details of the primary and redo operations are summarised in Tables 2 and 3. Reoperations revealed that the mesh was disconnected from the sacrum in 13 (30.2%) patients and from the rectal wall in 10 (23.3%) patients. The mesh was assessed to be overly proximally located in nine (20.9%) cases. In five patients, no specific reasons for recurrence were found during the redo operation. In most of these revisional operations (27/43; 62.8%), the previously used mesh was left in situ and a new one was placed after correction of rectal anatomy. Mere re-fixation of the proximal part of the mesh was used in only one patient. The whole mesh placed in primary rectopexy was removed (Figs. 1, 2) in three (7%) reoperations, and partial mesh removal was done in nine cases (20.9%). In one case of completely removed previous mesh, the suspected lesion in the rectal serosa was sutured intraoperatively. Polyester mesh was most often used in both primary operations (71.1%) and reoperations (55.8%). No biological grafts were implanted. The mean operating time was 157 (SD 46) minutes. Conversion to laparotomy occurred in one (2.3%) operation, which was performed using a laparoscopic technique. Intraoperative bleeding was minor. The ERP and IRP groups did not differ in terms of revisional surgery perioperative data.

**Table 2:** Primary operation details

	Data	*n*
Predominant symptom^a^ [*n* (%)]		
Prolapse without functional symptoms	11 (25.6)	22
Prolapse with incontinence	1 (2.3)	22
ODS only	6 (14.0)	22
Incontinence only	1 (2.3)	22
ODS with incontinence	3 (7.0)	22
Operation technique [*n* (%)]		
Laparoscopic	42 (97.7)	43
Open surgery	1 (2.3)	43
Mesh^b^ [*n* (%)]		
Polyester	27 (71.1)	38
Polypropylene and poliglecaprone	6 (15.8)	38
Polypropylene	5 (13.2)	38

Nominal variables are presented as counts and percentages (in parentheses). Continuous variables are presented as means and standard deviations

*ODS* obstructed defecation syndrome

^a^Data were available in 22 cases

^b^Data were available in 38 cases

**Table 3:** Perioperative data

	All (*n* = 43)	ERP (*n* = 22)	IRP (*n* = 21)	*p*
Operation technique [*n* (%)]				0.70
Laparoscopic	35 (81.4)	17 (77.3)	18 (85.7)	
Robotic	8 (18.6)	5 (22.7)	3 (14.3)	
Operating time [min, mean (SD)]	157 (46)	152 (49)	161 (43)	0.51
Operating theatre time [min, mean (SD)]	198 (43)	194 (49)	205 (35)	0.61
Bleeding [ml, mean (SD)]	51 (102)	43 (46)	60 (140)	0.60
Conversion [*n* (%)]				0.38
Yes	1 (2.3)	0 (0)	1 (4.8)	
No	42 (97.7)	22 (100)	20 (95.2)	
Primary finding during operation [*n* (%)]				> 0.9
Mesh off from sacral attachment area	13 (30.2)	5 (22.7)	8 (38.1)	
Mesh off from rectal attachment area	10 (23.3)	6 (27.3)	4 (19.0)	
Mesh off from pelvic attachment area	3 (7.0)	2 (9.1)	1 (4.8)	
Mesh positioned too proximally	9 (20.9)	5 (22.7)	4 (19.0)	
Mesh too loose	2 (4.7)	1 (4.5)	1 (4.8)	
No obvious reason	5 (11.6)	3 (13.6)	3 (14.3)	
Operation details [*n* (%)]				0.53
Old mesh removal, new mesh	3 (7.0)	2 (9.1)	1 (4.8)	
New fixation of proximal part	1 (2.3)	0 (0)	1 (4.8)	
Combined operation with dorsal rectopexy	3 (7.0)	1 (4.5)	2 (9.5)	
Old mesh left, new mesh	27 (62.8)	16 (72.7)	11 (52.4)	
Old mesh partly left, new mesh	9 (20.9)	3 (13.6)	6 (28.6)	
Mesh [*n* (%)]				0.15
Polyester	24 (55.8)	13 (59.1)	11 (52.4)	
Titanized polypropylene mesh	12 (27.9)	8 (36.4)	4 (19.0)	
Polypropylene and poliglecaprone	3 (7.0)	0 (0)	3 (14.3)	
Caudal fixation of mesh [*n* (%)]				> 0.9
Through the pelvic floor	7 (16.3)	4 (19.0)	3 (15.8)	
To levator muscles	31 (72.1)	16 (76.2)	15 (78.9)	
To rectum only	2 (4.7)	1 (4.8)	1 (5.3)	
Number of stiches to rectum [*n* (%)]				0.14
0–4	19 (44.2)	8 (36.4)	11 (52.4)	
5–6	16 (37.2)	9 (40.9)	7 (33.4)	
7–14	8 (18.7)	5 (22.7)	3 (14.4)	
Vaginal stiches were used [*n* (%)]	33 (76.7)	14 (63.6)	19 (90.5)	0.13
Suturing technique [*n* (%)]				0.73
Intracorporeal	10 (23.3)	6 (27.3)	4 (19.0)	
Extracorporeal	29 (67.4)	15 (68.2)	14 (66.7)	

Nominal variables are presented as counts and percentages (in parentheses). Continuous variables are presented as means and standard deviations

*ERP* external rectal prolapse, *IRP* internal rectal prolapse

Short-term postoperative outcomes are presented in Table 4. Clinical postoperative follow-up information was available for all patients. A total of ten (23.3%) patients experienced complications, of which two (4.7%) were mesh erosions through vaginal wall identified 1 month and 9 months after the redo VMR. They occurred in patients, in whom the previously used mesh was left in situ. In one case, the protruded part of a mesh was resected transvaginally and the defect was sutured. In the second case, first a transvaginal defect suturing was carried out, followed by a mesh removal and vaginal wall defect suturing laparoscopically. There were 3 (7%) vaginal perforations. In two of these cases, the vaginal wall was sutured intraoperatively. In one case, the small 2–3 mm size vaginal wall defect was noticed 13 days postoperatively with haematoma between the vagina and rectum requiring only conservative treatment. There were three cases of bleeding, of which one required postoperative laparoscopic haematoma evacuation. Of all complications, two cases were Clavien–Dindo grade ≥ 3 events. There was no postoperative 60-day mortality. A recurrence of the prolapse was noticed only in one patient, giving an overall recurrence rate of 4.5% for ERP after redo VMR. The symptoms caused by this minor prolapse were so mild that there was no need for any action. Three (7.0%) patients were reoperated on for various reasons. One patient was treated with sphincteroplasty for lasting anal incontinence, and for one patient, transanal haemorrhoidal dearterialization (THD) was done due to mucosal prolapse after redo RVMR for ERP. A third reoperation was posterior colporrhaphy, for which the indication was residual rectocele after IRP repair by LVMR. The median in-hospital stay was 3.7 days (range 1–12 days). Patients with IRP faced more complications (38.1% vs. 9.1%, *p* = 0.03) than patients with ERP. In other respects, the subgroups did not differ in terms of short-term postoperative outcome.

**Table 4:** Short-term postoperative outcomes

	All (*n* = 43)	ERP (*n* = 22)	IRP (*n* = 21)	*p*
In-hospital stay [days, mean (SD)]	3.7 (2.3)	3.8 (2.6)	3.6 (1.8)	0.71
(Range)	(1–12)	(1–12)	(2–9)	
Complication [*n* (%)]	10 (23.3)	2 (9.1)	8 (38.1)	0.03
Bleeding	3 (7.0)	1 (4.5)	2 (9.5)	
Vaginal perforation	3 (7.0)	1 (4.5)	2 (9.5)	
Mesh-related complication	2 (4.7)	0 (0)	2 (9.5)	
Paralytic ileus	1 (2.3)	0 (0)	1 (4.8)	
Small bowel obstruction	1 (2.3)	0 (0)	1 (4.8)	
Clavien–Dindo ≥ 3 complications	2 (4.7)	0 (0)	2 (9.5)	0.23
Reoperation [*n* (%)]				> 0.9
Sphincteroplasty	1 (2.3)	0 (0)	1 (4.8)	
Posterior colporrhaphy	1 (2.3)	0 (0)	1 (4.8)	
Transanal hemorrhoidal dearterialization	1 (2.3)	1 (4.5)	0 (0)	

Nominal variables are presented as counts and percentages (in parentheses). Continuous variables are presented as means and standard deviations

*ERP* external rectal prolapse, *IRP* internal rectal prolapse

The patient-reported long-term functional outcome and QoL data are presented in Table 5. The median length of follow-up was 3.0 years (SD 1.2, range 1.6–6.4 years). One patient died during the follow-up time for reasons unrelated to operative treatment. The response rate was 59.5% (25/42 living patients). As the comparable postoperative functional data after primary operation was accessible in 16 (37.2%) patients and only 10 (23.8%) of them responded to questionnaires after the redo operation, it was not possible to evaluate the individual benefit of the reoperation for all patients. Of all patients, 65.2% reported postoperative defecatory symptom relief. The mean Wexner score was 6 (SD 5) and the mean ODS score was 16 (SD 9). Of all patients, 9.3% reported significant ongoing incontinence symptoms and 16.3% ongoing ODS. The Wexner scores, ODS scores and the reported postoperative discomfort with incontinence or obstructed defecation did not differ between the ERP and IRP groups. Also, the effect on sexual life was comparable between the groups. The patients with ERP reported significantly better postoperative VAS scale scores in the effect on QoL. Also, symptom changes towards the better or symptom relief results on VAS scale scores were better in patients with ERP than in IRP patients. De novo urinary incontinence and urinary retention were comparable between the ERP and IRP groups. More patients with IRP reported de novo pelvic pain compared to the ERP group. In all, 13/24 (54.2%) of respondents were subjectively satisfied with the redo LVMR or RVMR result (25.0%), were dissatisfied and (20.8%) patients could not say. Of the ERP and IRP patients, 69.2% and 36.4% of respondents, respectively, were satisfied with the redo LVMR or RVMR results, with no statistical significance.

**Table 5:** Long-term functional outcome and quality of life

	All patients		ERP		IRP		*p*
	Data	*N*	Data	*n*	Data	*n*	
Follow-up time, years^a^, [mean (SD)] Range	3.0 (1.2) (1.6–6.4)	42	2.9 (1.3) (1.6–6.4)	22	3.2 (1.2) (1.6–5.9)	20	0.65
Questionnaire response rate							
After redo operation [*n* (%)]	25 (59.5)	42	13 (59.1)	22	12 (60.0)	20	> 0.9
Wexner score (0–20) [mean (SD)]^b^	6 (5)	23	7 (6)	12	5 (4)	11	0.39
Wexner score > 9 [*n* (%)]^c^	4 (9.3)	23	2 (9.1)	12	2 (9.5)	11	> 0.9
Incontinence discomfort on VAS (0–100) [mean (SD)]^d^	30 (32)	20	29 (33)	10	31 (33)	10	0.89
ODS score (0–40) [mean (SD)]^e^	16 (9)	24	13 (8)	12	19 (9)	12	0.10
ODS score > 20 [*n* (%)]^f^	7 (16.3)	24	2 (9.1)	12	5 (23.8)	12	0.37
Obstructed defecation discomfort on VAS (0–100) [mean (SD)]^d^	63 (31)	24	57 (33)	12	70 (28)	12	0.29
Symptom change (before vs postoperatively) on VAS (0–100) [mean (SD)]^g^	72 (24)	23	88 (14)	11	58 (22)	12	0.001
Symptom relief (VAS 61–100) [*n* (%)]	15 (65.2)	23	10 (90.9)	11	5 (41.7)	12	0.027
Effect on QoL on VAS (0–100) [mean (SD)]^g^	69 (25)	24	83 (16)	12	55 (25)	12	0.003
Improved QoL (VAS 61–100) [*n* (%)]	15 (62.5)	24	10 (83.3)	12	5 (41.7)	12	0.089
Effect on sexual life on VAS (0–100) [mean (SD)]^g^	60 (32)	7	52 (41)	4	71 (18)	3	0.49
De novo urinary incontinence [*n* (%)]	8 (32.0)	25	4 (30.8)	13	4 (33.3)	12	> 0.9
De novo urinary retention [*n* (%)]	9 (36.0)	25	5 (38.5)	13	4 (33.3)	12	> 0.9
De novo pelvic pain [*n* (%)]	9 (36.0)	25	2 (15.4)	13	7 (58.3)	12	0.041
Satisfaction with redo operation							0.11
No [*n* (%)]	6 (25.0)	24	1 (7.7)	13	5 (45.5)	11	
Yes [*n* (%)]	13 (54.2)	24	9 (69.2)	13	4 (36.4)	11	
Cannot say [*n* (%)]	5 (20.8)	24	3 (23.1)	13	2 (18.2)	11	

Nominal variables are presented as counts and percentages (in parentheses). Continuous variables are presented as means and standard deviations

*ERP* external rectal prolapse, *IRP* internal rectal prolapse

^a^Time from redo operation to questionnaire response date

^b^Wexner score for faecal incontinence (min–max; 0–20)

^c^Wexner > 9: significant ongoing obstructed defecation symptoms

^d^VAS visual analogue scale (no discomfort–great discomfort; 0–100)

^e^ODS: Obstructed Defecation Symptom score (min–max; 0–40)

^f^ODS > 20: significant ongoing obstructed defecation symptoms

^g^VAS: visual analogue scale (much worse–much better; 0–100)

## Discussion

To our knowledge, this is the first study reporting exclusively the outcomes of redo LVMR and RVMR. Further, although the number of patients in our study is limited, it is the largest study to focus on the surgical treatment of recurrent ERP and IRP. The results provide new information about the indications, intraoperative findings and technical details of redo surgery after failed primary minimally invasive VMR. The present study shows that minimally invasive redo VMR appears to be a safe and effective procedure for treating recurrent ERP and symptomatic IRP patients and has low recurrence and reoperation rates. Its effect on improving QoL, symptom changes for the better and less postoperative pelvic pain were more likely in patients with ERP than in IRP patients.

There are only two previous publications reporting the results of LVMR or RVMR performed as revisional surgery [15, 16]. They differ significantly from our study, since the primary operations in these studies varied, including perineal operations such as Altemeier and Delorme procedures. Further, the abdominal rectopexy operations in these series are not described in detail. Our study is unique since all the primary operations in our study were performed using the VMR technique. These differences make it difficult to compare our results to those of the previous studies. However, the overall results are well comparable. In most operations (62.8%) in our current study, the previously used mesh was left in situ and a new one was placed. Because there are no recommendations about the handling of previously placed mesh, we decided to leave it in situ to avoid complications related to the detaching of the mesh. In light of the results of our study, this seems to be a justified decision. There is only one systematic review on reoperations after failed rectal prolapse surgery. This review demonstrated the lack of evidence for recommendations about redo surgery after LVMR due to the absence of any supporting data [13]. As long as the debate about the best procedure for full rectal prolapse among coloproctologists continues, it is impossible to acquire guidelines for revisional surgery after recurrence. Currently, there is no evidence to support clinical decision-making regarding treatment of failed LVMR. However, our study indicates that redo LVMR and RVMR are valid options.

Previous VMR induces adhesion formation to the pelvic area, and it could be expected that this hampers reoperations. In light of this, our study showed a reasonably low complication rate of 23.3%, which is in line with the results of Gurland et al. [15], who reported a complication rate of 19.4%. Brunner et al. [16] had a lower complication rate (13.3%) in their study. It is noteworthy that almost all the patients in these previous series [15, 16] were initially operated on using other techniques than LVMR. In the work of Brunner et al., the patients were primarily operated on using a perineal approach, and thus the anatomical planes in the abdomen and pelvis were untouched. In addition, biological mesh was used in all operations performed in the study by Brunner et al. and in half of the operations in the study by Gurland et al. However, although revisional operations are presumably complex, the complication rate in our study was not higher than in the previously reported rate (up to 23.5%) for after primary operations [20].

In our current series, the reasonably low number of mesh-related complications (4.7%) is comparable to results reported after primary surgery (0–6.7%) [6, 20–22]. In previous American and German studies, the authors did not report any mesh-related complications [15, 16]. The reason for this may be a relatively short follow-up time in the Gurland study and the use of biological mesh by the Brunner group.

The recurrence rate in our study was relatively low, 4.5% for ERP after redo VMR. We have previously reported long-term recurrence rates of 7.1% for ERP and 6.1% for patients primarily operated on for IRP [6]. The corresponding recurrence rates of the observational Dutch and Belgian multicentre trials were 8.2% and 14.2%, respectively [20, 7].

Our study showed a median 3.7 day in-hospital stay, which does not differ from our previously published results of 501 primary LVMRs, where the median length of stay was 4 days [6]. Nowadays, however, the continuing process in the development of enhanced recovery protocols with reduced use of epidural analgesia in colorectal surgery and LVMR has shortened the in-hospital stay to as short as 1–2 days.

In our study, patients with ERP had a greater benefit from the redo operation than did the patients with IRP in terms of fewer complications, better postoperative-reported VAS scale scores in QoL (mean 83 vs mean 55, *p* = 0.003), better VAS scale scores in postoperative symptom relief (90.9% vs 41.7%, *p* = 0.027) and less reported de novo pelvic pain (15.4% vs 58.3%, *p* = 0.041). Neither the American [15] nor the German [16] studies included a comparison between ERP and IRP. Our current results are similar to those found in our earlier study [6], where ERP patients reported more symptom relief (85.9%) than IRP patients (68.4%, *p* < 0.001). In addition, a change for the better in QoL was seen more in ERP patients (84.9%) than in IRP patients (65.5%). However, instead of pelvic pain, the IRP patients more often reported the sensation of urgency (29.5%) compared to ERP patients [6].

The results of this study should be interpreted in light of some limitations, which are the small number of patients, retrospective data collection, lack of data concerning primary surgery and a quite low questionnaire response rate. Although the data were collected from three relatively large tertiary pelvic floor centres, we found only 43 reoperations. Patients are referred from all over Finland to these centres, which may be the reason for missing preoperative data and may also be a cause for the low response rate. Regrettably, as we had limited initial preoperative functional data, it was not possible to evaluate the individual benefit of the operation for all patients. Reoperations were performed as recommended [14], in tertiary centres after full reassessment by experienced surgeons operating approximately 50 VMR cases per year; however, these were done without any evidence-based guidelines. It would be of interest to know whether LVMR or RVMR is a better technique for redo surgery. However, due to the low number of patients and limited functional data our study could not clarify the issue. However, the outcomes of this study reflect normal clinical practice in the participating referral hospitals and indicate that there is a need for further studies to obtain evidence for clinical decision-making.

## Conclusions

Minimally invasive redo VMR appears to be a safe and effective procedure in treating recurrent ERP and symptomatic IRP patients with low recurrence and reoperation rates. Possibly patients with ERP may benefit more from the operation in terms of better QoL effect, better symptom relief effect and less reported postoperative pelvic pain.
